# Hepatitis C virus genotype and subtype distribution among ethnic minorities in Liaoning Province of China

**DOI:** 10.1097/MD.0000000000024137

**Published:** 2021-01-15

**Authors:** Rongkuan Li, Ying Xie, Wenzhi Liu, Yu Ma

**Affiliations:** a Department of Infectious Disease, The Second Affiliated Hospital of Dalian Medical University, Dalian; b Medical University, Dalian; b Department of Clinical Laboratory, Anshan Central Hospital, Anshan, Liaoning; Province, China.

**Keywords:** China, ethnic minorities, genotype, hepatitis C virus, Liaoning Province

## Abstract

To provide information and a basis for improved hepatitis C prevention and treatment, we aimed to determine the distribution of hepatitis C virus (HCV) genotypes among patients with hepatitis C from 4 ethnic minorities in Liaoning Province of China over the past 8 years and analyze and explore the virus’ genotype evolution and possible clinical significance.

For gene-sequencing, we collected peripheral blood samples of HCV-infected patients belonging to the Korean, Hui, Mongol, and Manchu ethnic minorities in Liaoning Province who were diagnosed at the Second Hospital of Dalian Medical University, Anshan Central Hospital, and the Second People's Hospital of Fuxin City between November 2011 and November 2019. To analyze genotype evolution and possible influencing factors, we determined the ratio of various genotypes. Among the 102 HCV-infected patients from 4 ethnic minorities in Liaoning Province, 46 had gene typing (GT)1b (45.10%), 15 had GT2a (14.71%), 14 had GT3a (13.73%), 13 had GT6a (12.75%), 3 had GT1a (2.94%), and 11 had an unclassified genotype (10.78%). The distribution of various genotypes in the Korean, Mongol, and Manchu ethnic minorities was significantly different (χ^2^ = 10.788, *P* = .029; χ^2^ = 7.846, *P* = .049; and χ^2^ = 22.400, *P* = .000, respectively). All ethnic minorities exhibited >40% of GT1b. In the Korean (14/33) and Manchu (14/30) ethnic minorities, the proportion of GT1b was significantly higher than those of other genotypes (*P* < .05). The ethnic Koreans had a high proportion of GT3a (18.18%, 6/33), whereas the ethnic Mongolians had a high proportion of GT6a (23.08%, 6/26). GT1a was only found in the Korean (6.06%, 2/33) and Manchu (3.33%, 1/30) ethnic minorities; in the Hui ethnic minority, only 3 genotypes were prevalent: GT1b, GT2, and GT3a. The ethnic minorities in Liaoning Province currently have diverse HCV genotypes; the most prevalent genotype is GT1b, followed by GT2a and GT3a, and the prevalence of GT3 and GT6 has increased. The distribution of HCV genotypes varies across different ethnic minorities. The Korean and Manchu ethnic minorities have the most prevalent genotypes, whereas the Hui ethnic minority has a relatively single distribution of the HCV genotype.

## 1 Introduction

Hepatitis C is a global epidemic. People of different sexes, ages, races, and ethnic groups are susceptible to hepatitis C virus (HCV) infection. The prevalence rate of HCV infection varies considerably across different regions and countries. In most cases of HCV infection, symptoms do not manifest at the acute and early stages of infection, and only <25% of patients with acute hepatitis C exhibit apparent clinical symptoms. According to the statistics of World Health Organization in 2017, approximately 71 million individuals had chronic HCV infection worldwide, with approximately 10 million of them being in China. Furthermore, mortality caused by HCV-induced liver cirrhosis and liver cancer accounts for approximately 20% of the global mortality.^[1,2]^ HCV is an RNA virus with a high degree of genetic variation. On the basis of the degree of genetic variation, HCV can be divided into at least 7 genotypes and 67 identified and 20 undefined gene subtypes based on the Simmonds genotyping system.^[3]^ The HCV genotypes vary greatly across different countries and regions. Therefore, they can be used for epidemiological investigation of HCV infection.

A recent meta-analysis assessing 168 records showed that the predominant HCV genotypes in China were the subtypes 1b and 2a and the distributions of HCV subtypes were different in the western and southern parts of the country. The prevalence of the subtypes 3a, 3b, and 6a was significantly increased, along with a decrease in the prevalence of subtypes 1b and 2a. Various HCV subtypes were identified among injection drug users, including the subtypes 3a, 3b, and 6a.^[4]^ Antiviral therapy for chronic HCV infection has entered the era of direct antiviral agents (DAAs), which inhibit the virus by directly inhibiting HCV protease, RNA polymerase, or other viral enzymes. The new oral anti-HCV drugs approved for clinical application or those in phase II and III clinical trials mainly include NS3/4A protease inhibitors (PIs), NS5A inhibitors, and NS5B polymerase inhibitors (PIs).^[5]^ It is preferable to recommend DAA treatment without interferon, which can help achieve >90% sustained virological response (SVR) in patients with known major genotypes and minor genotypes.^[8]^ Genotyping plays an important role in the epidemiological investigation of HCV; study of virulence; development of specific vaccines for different subtypes of viruses; and study of the natural course of viral infection and its role in the pathogenesis of persistent infection, viral evolution, host–virus interaction, and clinical therapeutic effect. Therefore, HCV genotyping has attracted wide attention in recent years.

China has recently proposed the cancellation of the comprehensive national plan for HCV infection and instead planned to establish a public health policy based on local epidemiology, healthcare infrastructure, local screening, treatment, prevention coverage, and available financial or human resources.^[6]^ Liaoning Province has a large population of ethnic minorities in China. In addition to the Han, 51 ethnic minorities, including Manchu, Mongol, Hui, Korean, and Xibo, are known to live in Liaoning Province. In terms of the absolute population of ethnic minorities, Liaoning Province ranks fifth among provinces in China; however, data on the genotype distribution of HCV among HCV-infected patients among the major ethnic minorities in Liaoning Province are not available. We conducted quantitative detection and gene-sequencing typing of hepatitis C virus ribonucleic acid (HCV-RNA) in the peripheral blood samples of patients from the Korean, Hui, Mongol, and Manchu ethnic minorities diagnosed with hepatitis C between 2011 and 2019. These samples were used to analyze and compare the distribution of and determine the differences in genotypes among the different ethnic minorities.

## 2 Methods

### 2.1 Study subjects

This study enrolled 102 patients from different ethnic minorities who were diagnosed with hepatitis C (with positive serum anti-HCV) at the Second Hospital of Dalian Medical University, Anshan Central Hospital, and the Second People's Hospital of Fuxin City between November 2011 and November 2019. Investigators obtained informed consent before enrolling participants into this study. All patients met the diagnostic criteria of the Guidelines for the Prevention and Treatment of Hepatitis C, which was formulated by the Chinese Society of Hepatology and Society of Infectious Diseases.^[7]^ The study was approved by the Research Ethics Committee of Dalian Medical University, Liaoning, China. All patients provided written informed consent.

### 2.2 Instruments and methods

We collected and centrifuged venous blood samples from all patients to obtain 1–2 mL of serum. To detect the HCV-RNA content, we used real-time quantitative polymerase chain reaction using the ABI 7500 platform (Applied Biosystems). Then we extracted HCV-RNA (viral RNA purification kit; Acon Biotech Co., Ltd.) and reverse-transcribed it into complementary deoxyribonucleic acid (ReverTra Ace q polymerase chain reaction [PCR] RT Kit, TOYOBO). Using complementary deoxyribonucleic acid as a template, the PCR primer sequences 5′-CGCAATGTGCGTAACGTCAACGATAC-3′ and 5′-GTAGGCGACGACTTCTTCAT CA-3′ were designed using the core/E1 region of the HCV genomic RNA to amplify the target gene using ABI PRISM 7300. The purified target gene product was sequenced using the Diagnostic Kit For Hepatitis C Virus Genotyping (PCR-sequencing Analysis) (Life-DaAn Diagnostic Products Technology Co., Ltd.) and ABI 3500 gene sequencer. The primer sequence is 5′-GCCGAGGTCATCCGCTACTT-3′. The sequencing results were analyzed using the Mutation Surveyor software. For typing comparison, the obtained sequences were submitted to the National Center for Biotechnology Information Genotyping interface.

### 2.3 Statistical processing

All statistical processing and analyses were performed using SPSS version 17.0 statistical software (International Business Machines Corporation, IBM). Enumeration data were expressed as mean ± standard deviation, and enumeration data and rate were compared using *χ*^2^ test. A sample size of <40 was determined using the Fisher exact test. *P* < .05 was considered significant.

## 3 Results

Among the 102 HCV-infected patients, 33 were ethnic Korean (32.35%), 13 were ethnic Hui (12.74%), 26 were ethnic Mongol (25.49%), and 30 were ethnic Manchu (29.41%) individuals. Table 1 summarizes patients’ details. Overall, 91 patients were successfully genotyped, with a genotyping rate of 82.42%. We detected 5 genotypes: gene typing (GT)1a, GT1b, GT2a, GT3a, and GT6a; 3 patients had GT1a (2.94%), 46 had GT1b (45.10%), 15 had GT2a (14.71%), 14 had GT3a (13.73%), 13 had GT6a (12.75%), and 11 had an unclassified genotype (10.78%).

**Table 1:** General information on the population with hepatitis C in the 4 major ethnic minorities in Liaoning Province.

Minority	Sex (male/female)	Age (yr)	History of surgery (n, %)	History of blood transfusion (n, %)	Clinical symptoms (n, %)	Abnormal liver function (n, %)	HCV RNA >1 × 103 IU/mL (n, %)
Korean	19/14	12–68	21 (63.64)	24 (72.73)	22 (66.67)	24 (72.73)	31 (93.94)
Hui	7/6	27–71	9 (69.23)	9 (69.23)	10 (76.92)	10 (76.92)	3 (23.08)
Mongolian	15/11	15–96	10 (38.46)	16 (61.54)	14 (53.85)	18 (69.23)	25 (96.15)
Manchu	16/14	19–78	12 (40.00)	14 (46.67)	19 (63.33)	16 (53.33)	27 (90.00)
Total	57/45	12–96	52 (50.98)	63 (61.76)	65 (63.73)	20 (19.61)	93 (91.18)

**Figure 1:**
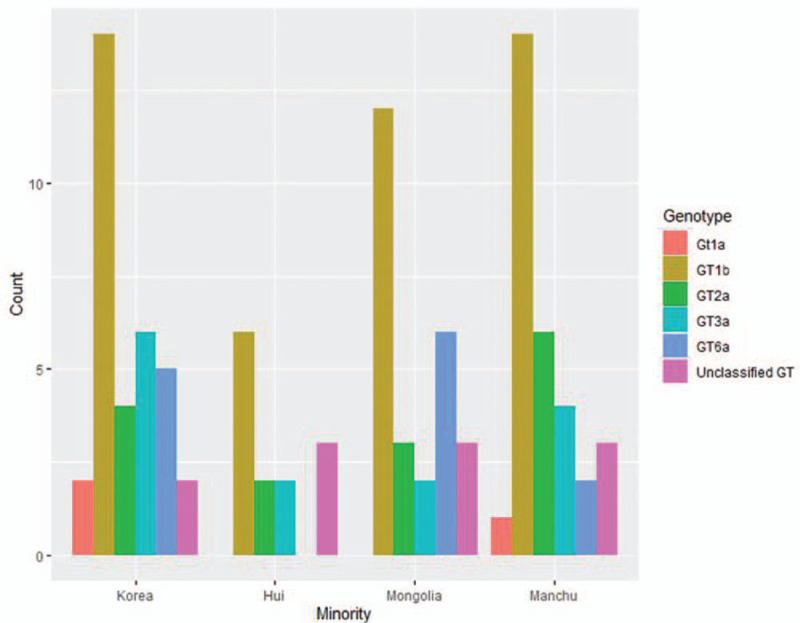
Distribution of HCV genotypes in 4 ethnic minority hepatitis C patients in Liaoning Province (n, %).

The distribution of different genotypes among the Korean, Mongol, and Manchu ethnic minorities showed significant differences (χ^2^ = 10.788, *P* = .029; χ^2^ = 7.846, *P* = .049; and χ^2^ = 22.400, *P* = .001, respectively). GT1b was observed at significantly higher rate than other genotypes among the Korean (14/33) and Manchu ethnic minorities (*P* < .05). Moreover, the Korean ethnic minority exhibited a higher proportion of GT3a than the other ethnic minorities (18.18%); however, the difference was not significant according to Fisher's exact text (*P* > .05). Meanwhile, the Mongol ethnic minority exhibited a higher proportion of GT6a than the other ethnic minorities (23.08%), with no significant difference (*P* > .05). GT1a had the lowest rate, with only 2 cases in the Korean (6.06%, 2/33) and 1 in the Manchu (3.33%, 1/30) ethnic minorities. In the Hui ethnic minority, only 6 patients had GT1b (46.15%), 2 had GT2a (15.38%), and 2 had GT3a (15.38%) (Fig. 1). Because of the limited sample size, GT1a was not found in the Hui and Mongolian ethnic minorities and GT6a was not found in the Hui ethnic minority.

## 4 Discussion

With the advancement of diagnostic technology and clinical drug treatment, an increasing number of patients have benefited from timely, accurate, and effective diagnosis and treatment for hepatitis C. The treatment plan for HCV infection depends on virus genotyping, and HCV genotyping is closely related to clinical outcomes and antiviral effects. Moreover, HCV genotyping helps in understanding the evolution and distribution of the virus and is essential for future HCV infection prevention and treatment. Considering the lack of an effective vaccine for HCV infection prevention, determining the distribution characteristics of HCV genotypes and transmission modes of HCV is critical to control the spread of HCV infection. In future, genotype-specific protocols will continue to be recommended for clinical use. The main consideration is that the affordability of HCV genotyping in China is better than that of the pan genotype program; moreover, it is affordable in certain populations (such as patients with decompensated cirrhosis, children/adolescents, and patients with kidney injury).^[8]^

This study reports the distribution of HCV genotypes among ethnic minorities in Liaoning Province. The results showed that 5 genotypes, namely, GT1b (45.10%), GT2a (14.71%), GT3a (13.73%), GT6a (10.78%), and GT1a (3 cases, 2.94%), are prevalent among the Korean, Hui, Mongol, and Manchu minorities in Liaoning Province. In China, the prevalent genotypes are Ib and 2a, which are predominant in the north. According to the present study findings, ethnic minorities in Liaoning Province exhibit basically the same distribution of HCV genotypes as that exhibited by the Han, but the expression levels of GT3 and GT6 are higher than the domestic average (GT3 and GT6 account for 9.10% and 6.30%, respectively, at the domestic level).^[9]^ This is attributable to the limited sample size of patients with hepatitis C among the ethnic minorities; it may also be related to the different characteristics and lifestyles of the ethnic groups. The results showed a larger proportion of GT1b (14/33) than those of other genotypes in HCV-infected ethnic Korean patients (*P* < .05). Using the probe reverse hybridization assay, Zhongxie et al. found that GT1b is the dominant genotype among ethnic Korean patients with hepatitis C,^[10]^ which is consistent with the present study results; however, they only detected GT1b and GT2a, possibly owing to the fewer genotypes included in the probe reverse hybridization technology. Therefore, the possibility of presence of other genotypes cannot be excluded. In our study, 5 genotypes were identified in the Korean ethnic minorities, of which GT3a and GT6a both accounted for >10%; in particular, GT3a (18.18%) was found at a relatively higher rate. The proportion of patients with GT3 is much higher in China than in European and American countries. There are limited data showing that the SVR rate of sofosfovir/vapatavir in Chinese patients with genotype 3B without cirrhosis after 12 weeks of treatment is 96%, and the SVR rate of patients with liver cirrhosis is 50%; therefore, in areas where the prevalence rate of genotype 3B subtype is >5%, it is necessary to identify gene 3b subtype.^[15]^ In the 26 HCV-infected patients of the Mongol ethnic minorities, GT1b (12/26) accounted for the highest proportion (46.15%), followed by GT3a and GT6a (which accounted for >10%); furthermore, GT6a (23.08%) was found at a higher rate than GT3a (11.54%). HCV genotyping in the Mongol ethnic minorities has been seldom reported. Nuenjiya used polymerase chain reaction–restriction fragment length polymorphism to detect HCV genotypes in Inner Mongolia and Mongolia^[11]^ and found that GT1b was the dominant genotype. This result is consistent with our study findings. Our study results showed that, in addition to the high proportion of GT1b and GT2a, GT3a and GT6a accounted for a large proportion. The 13 HCV-infected patients in the Hui ethnic minority exhibited a relatively single distribution of HCV genotype. Unlike previous reports,^[12]^ the present study only detected 6 GT1b cases (46.15%), 2 GT2a cases (15.38%), and 2 GT3a cases (15.38%), whereas GT3c was not detected. We detected 5 genotypes in the HCV-infected Manchu patients, among which GT1b (14/30) accounted for a significantly larger proportion (46.67%) than other genotypes (*P* < .05), followed by GT2a (6/30, 20%); these findings are consistent with those of a study reporting that lb and 2a are the predominant genotypes in the Han population in northern China.^[13]^ Overall, our study results showed that the HCV genotypes among the ethnic minorities in Liaoning Province are diverse, with GT1b being the most prevalent, followed by GT2a and GT3a. The GT4, GT5, and GT7 genotypes were not detected, and the prevalence of GT3 and GT6 was found to be increased. The distribution of HCV genotypes varies across different ethnic minorities. The Korean and Manchu ethnic minorities have the most prevalent genotypes, whereas the Hui ethnic minority has a relatively single distribution of the HCV genotype.

Although the ethnic minorities in China represent a small population group, they are widely distributed. Regarding the distribution characteristics of these ethnic groups, different ethnic groups reside together, whereas some reside in individual concentrated communities. The ethnic minorities as well as the Han population reside together in some areas. This distribution pattern has been formed by the interaction and exchange among different ethnic groups during the course of historical development. The HCV genotype 6 may be the oldest HCV genotype.^[14]^ Nie et al. also found that the HCV subtypes in mainland China are still dominated by 1b,^[15]^ but the proportion of 6a has exceeded that of 2a, and 6a has become the second largest subtype; the proportion of the subtype 3a has been increasing, along with a significant increase in the proportions of other subtypes. In the present study, the sample size for identifying the HCV genotypes in the ethnic minorities of Liaoning Province was limited. Most ethnic minorities reside in more remote areas; their willingness to seek medical treatment is weak and economic condition is poor, leading to limitations of sample collection in our study. Additionally, although the detection reagent covers most genotypes, rare types cannot be detected, leading to a possible bias or imprecision in the study results. In future studies, larger sample sizes are needed to observe changes in genotype distribution to provide a basis for the development of diagnostic reagents, vaccines, and antiviral treatment strategies against HCV infection in Liaoning Province.

## Acknowledgments

We gratefully acknowledge the valuable cooperation of Yang Song (Science and Education Department of Anshan Central Hospital) in preparing this application note.

## Author contributions

Ying Xie and Rongkuan Li contributed to the conception of the study.

Ying Xie and Yu Ma significantly contributed to analysis and manuscript preparation.

Ying Xie and Rongkuan Li performed data analyses and wrote the manuscript.

Wenzhi Liu and Yu Ma helped perform the analysis with constructive discussions.

**Software:** Wenzhi Liu.

**Investigation:** Yu Ma.

**Writing – original draft:** Rongkuan Li.

**Writing – review & editing:** Ying Xie.

## Abbreviations

Abbreviations: GT = gene typing, HCV = hepatitis C virus, HCV-RNA = hepatitis C virus ribonucleic acid, PCR = polymerase chain reaction.
